# DNA damage markers in dermal fibroblasts *in vitro* reflect chronological donor age

**DOI:** 10.18632/aging.100890

**Published:** 2016-01-30

**Authors:** Mariëtte E.C. Waaijer, Eleonora Croco, Rudi G.J. Westendorp, P. Eline Slagboom, John M. Sedivy, Antonello Lorenzini, Andrea B. Maier

**Affiliations:** ^1^ Department of Gerontology and Geriatrics, Leiden University Medical Center, 2300 RC Leiden, the Netherlands; ^2^ Department for Life Quality Studies, University of Bologna, 40126 Bologna, Italy; ^3^ Department of Public Health and Center for Healthy Aging, Faculty of Health and Medical Sciences, University of Copenhagen, 1123 Copenhagen, Denmark; ^4^ Department of Molecular Epidemiology, Leiden University Medical Center, 2300 RC Leiden, The Netherlands; ^5^ Netherlands Consortium for Healthy Aging, Leiden University Medical Center, 2300 RC Leiden, The Netherlands; ^6^ Department of Molecular Biology, Cell Biology and Biochemistry, Brown University, Providence, RI 02903, USA; ^7^ Department of Biomedical and Neuromotor Sciences, University of Bologna, 40126 Bologna, Italy; ^8^ Department of Internal Medicine, Section of Gerontology and Geriatrics, VU University Medical Center, 1007 MB Amsterdam, The Netherlands; ^9^ Department of Medicine, Royal Melbourne Hospital, University of Melbourne, Parkville VIC 3050, Australia

**Keywords:** human aging, 53BP1, telomere-associated foci, micronuclei, biological age, 53BP1 foci: all foci within the entire nucleus, TAF - telomere-associated foci: the subset of 53BP21 foci that colocalized with telomeric DNA

## Abstract

The aging process is accompanied by an accumulation of cellular damage, which compromises the viability and function of cells and tissues. We aim to further explore the association between *in vitro* DNA damage markers and the chronological age of the donor, as well as long-lived family membership and presence of cardiovascular diseases. Therefore, numbers of 53BP1 foci, telomere-associated foci (TAF) and micronuclei were measured in cultured dermal fibroblasts obtained from three age groups of donors (mean age 22, 63 and 90 years). Fibroblasts were cultured without a stressor and with 0.6 μM rotenone for 3 days. We found that 53BP1 foci and TAF were more frequently present in fibroblasts of old donors compared to middle-aged and young donors. No association between micronuclei and donor age was found. Within the fibroblasts of the middle-aged donors we did not find associations between DNA damage markers and long-lived family membership or cardiovascular disease. Results were comparable when fibroblasts were stressed*in vitro* with rotenone. In conclusion, we found that DNA damage foci of cultured fibroblasts are significantly associated with the chronological age, but not biological age, of the donor.

## INTRODUCTION

The DNA damage theory of aging is based on the efficacy of maintenance and repair mechanisms to ensure longevity [1,2]. DNA damage which is not repaired can compromise the function and viability of the cell and therefore the integrity of tissues, eventually contributing to aging and a decreased lifespan. Cells with DNA damage foci, marked by DNA damage response (DDR) factors such as DDR mediator protein 53BP1 or phosphorylated H2AX, have been found more frequently in old compared to young animals [3]. Some of these DNA damage foci are localized specifically on telomeric DNA, so-called telomere associated foci (TAF), which have a higher prevalence at higher age in several animal tissues as well [4-7]. Furthermore, a higher number of micronuclei *in vitro*, as a marker of unresolved DNA damage and chromosomal instability, associates with a lower maximum lifespan in various mammal species [8].

Research on DNA damage and human aging is scarce, especially including extreme ages. Lymphocytes derived from older donors showed more DNA damage foci compared to young donors [9]. A similar trend was observed in cultured primary fibroblasts strains derived from donors of different ages, albeit in a very low number of strains (5 donors) [9]. In a recent study, numbers of 53BP1 foci in dermal fibroblasts were also positively associated with the age of the donor [10]. Micronuclei numbers were shown to be higher at higher age in lymphocytes (reviewed in) [11] and buccal cells [12]. However, an inverse relation between the number of 53BP1 foci and micronuclei has also been described between mouse and human cells [8].

In the present study we significantly extent the published literature on the relation of DNA damage foci and chronological age in human fibroblasts by a higher number of donors with a broad age range. Furthermore, we study if DNA damage is associated with biological age (membership of long-lived family and presence of cardiovascular and/or metabolic disease).

## RESULTS

Donor characteristics are shown in Table 1.

**Table 1:** Characteristics of the donors

	Young (N=10)	Middle-aged		Old (N=10)
		Offspring (N=40)	Partners (N=40)	
Demographic data				
Female, no.(%)	7 (70.0)	20 (50.0)	20 (50.0)	6 (60.0)
Age, years, mean (SD)	22.8 (1.5)	63.1 (7.1)	63.2 (7.6)	90.2 (0.5)
Anthropometric data, mean (SD)				
Body mass index, kg/m^2^	22.2 (1.8)^a^	26.8 (4.7)^b^	25.6 (3.4)^c^	25.4 (3.8)
Co-morbidities, no./total known (%)				
Myocardial infarction	0/10 (0.0)	0/37 (0.0)	0/38 (0.0)	3/10 (30.0)
Cerebrovascular accident	0/10 (0.0)	1/38 (2.6)	2/38 (5.3)	2/10 (20.0)
Hypertension	0/10 (0.0)	9/38 (23.7)	8/38 (21.1)	5/10 (50.0)
Diabetes mellitus	0/10 (0.0)	2/37 (5.4)	5/37 (13.5)	2/10 (20.0)
Malignancies	0/10 (0.0)	1/36 (2.8)	2/36 (5.6)	1/10 (10.0)
Chronic obstructive pulmonary disease	0/10 (0.0)	1/38 (2.6)	2/37 (5.4)	1/10 (10.0)
Rheumatoid arthritis	0/10 (0.0)	0/38 (0.0)	0/38 (0.0)	3/10 (30.0)
Intoxications, no./total known (%)				
Smoking, current	0/10 (0.0)	6/38 (15.8)	4/38 (10.0)	1/10 (10.0)

SD: standard deviation. a: N=8, b: N=38, c: N=39. Offspring: offspring of nonagenarian siblings, i.e. member of a long-lived family. Partners: the partners of the offspring of nonagenarian siblings.

### Association between DNA damage markers and chronological age

Table 2 shows the association of the percentages of fibroblast nuclei with ≥1/≥2 53BP1 foci, TAF and fibroblasts with ≥1/≥2 micronuclei with chronological age. In the non-stressed state, the percentages of nuclei with both ≥1 and ≥2 53BP1 foci per nucleus were positively associated with chronological age (both P<0.001). The percentage of nuclei with ≥1 TAF per nucleus was also positively associated with age (P=0.001), but the percentage of nuclei with ≥2 TAF per nucleus was not significantly associated (P=0.147). The percentages of cells with ≥1/≥2 micronuclei per cell were not associated with the age of the donor (P=0.711 and P=0.411 respectively).

**Table 2:** 53BP1 foci, TAF and micronuclei dependent on chronological age of the donor

	Chronological age	
	Slope (SE)	P-value
**Non-stressed state**		
% nuclei with ≥1 53BP1 foci/nucleus	0.23 (0.06)	<0.001
% nuclei with ≥2 53BP1 foci/nucleus	0.15 (0.04)	<0.001
% nuclei with ≥1 TAF/nucleus	0.16 (0.05)	0.001
% nuclei with ≥2 TAF/nucleus	0.05 (0.04)	0.147
% cells with ≥1 micronuclei/cell	0.00 (0.01)	0.711
% cells with ≥2 micronuclei/cell	0.00 (0.00)	0.411
**Rotenone stressed state**		
% nuclei with ≥1 53BP1 foci/nucleus	0.29 (0.07)	<0.001
% nuclei with ≥2 53BP1 foci/nucleus	0.16 (0.06)	0.005
% nuclei with ≥1 TAF/nucleus	0.16 (0.04)	<0.001
% nuclei with ≥2 TAF/nucleus	0.06 (0.02)	0.003
% cells with ≥1 micronuclei/cell	0.01 (0.02)	0.787
% cells with ≥2 micronuclei/cell	0.01 (0.01)	0.330
**Δ stressed and non-stressed state**		
% nuclei with ≥1 53BP1 foci/nucleus	0.06 (0.05)	0.243
% nuclei with ≥2 53BP1 foci/nucleus	0.01 (0.04)	0.796
% nuclei with ≥1 TAF/nucleus	0.00 (0.04)	0.904
% nuclei with ≥2 TAF/nucleus	0.00 (0.04)	0.945
% cells with ≥1 micronuclei/cell	−0.01 (0.02)	0.725
% cells with ≥2 micronuclei/cell	0.01 (0.01)	0.416

The slope of the adjusted regression line is given (linear mixed model, with adjustment for gender, batch and repeated measurements; independent variable: age in years, continuously). SE: standard error. TAF: telomere-associated foci. Δ: difference.

In the rotenone-stressed state, depicted in Table 2 as well, the percentages of nuclei with ≥1 and ≥2 53BP1 foci per nucleus were positively associated with age (P<0.001 and P=0.005 respectively). The percentages of nuclei with ≥1 and ≥2 TAF were also positively associated with age (P<0.001 and P=0.003, respectively). The percentage of cells with ≥1/≥2 micronuclei per cell were not significantly associated with age (P=0.787 and P=0.330). No associations of the differences between the stressed and non-stressed state of 53BP1 foci, TAF and micronuclei was found with age (all: P>0.05).

**Figure 1:**
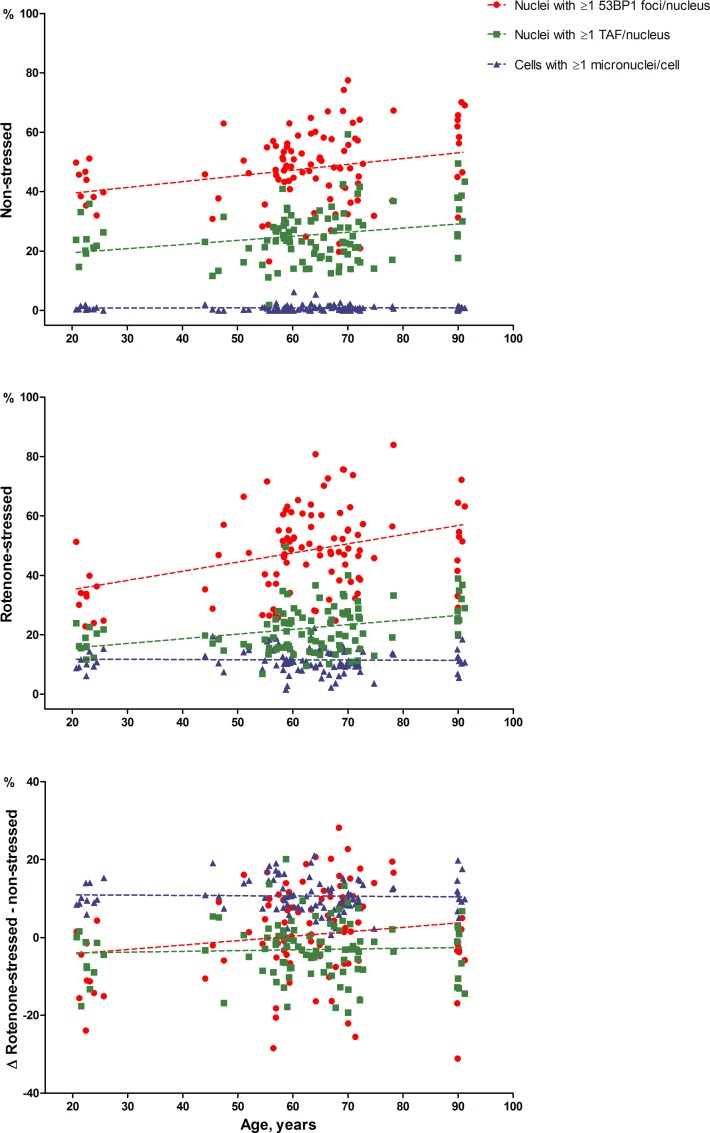
53BP1 foci, TAF and micronuclei dependent on chronological age The average percentage of nuclei with ≥1 53BP1 foci (circles) or TAF (squares) per nucleus and the average percentage of cells with ≥1 micronucleus (triangles) are depicted on the y-axis. Average percentages of duplicate series were used. Unadjusted regression lines are shown.

Figure 1 visualizes the associations between percentages of 53BP1 foci, TAF positive nuclei and percentages of micronuclei positive fibroblasts with chronological age. The data points and regression lines are shown separately for the non-stressed state, the rotenone-stressed state and the difference between rotenone-stressed and non-stressed state.

### Association between 53BP1 foci and micronuclei

**Figure 2:**
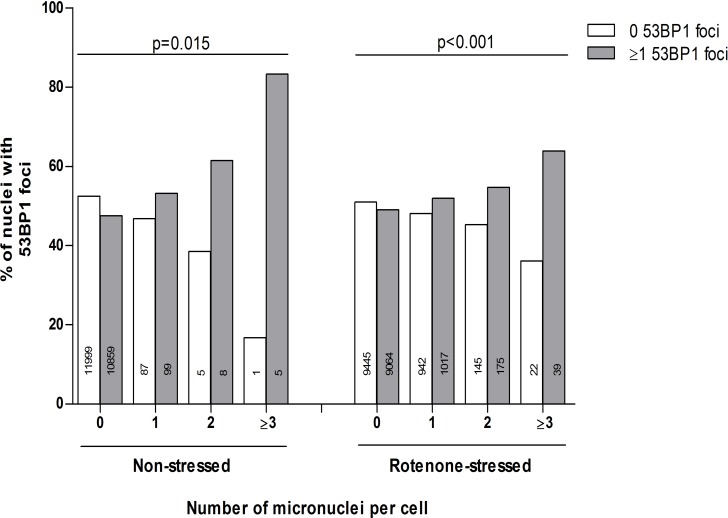
The association of the absolute number of micronuclei and 53BP1 foci Dependence of 0 or ≥1 53BP1 foci on individual micronuclei counts (both duplicate series). Micronuclei counts equal to or higher than 3 were combined in one category: ≥3. Linear by linear association tests were performed to test the linearity of differences in proportions.

Figure 2 shows the association of the absolute counts of 53BP1 foci and micronuclei on a single cell level. Most of the fibroblasts had 0 and some 1 micronuclei per cell. Cells with 2 or more micronuclei were rarely present. The linear by linear association of the distribution of 53BP1 foci and micronuclei count categories was significant in both non-stressed control state and rotenone-stressed state, with more frequent presence of ≥1 53BP1 foci in cells with more micronuclei (P=0.015 and P<0.001, respectively).

### Association between DNA damage markers and biological age

The association of DNA damage markers and biological age within the middle-aged group is shown in Supplementary Tables 1 and 2. No significant differences in average percentage of nuclei with ≥1/≥2 53BP1 foci, TAF or micronuclei were observed between offspring and partners and those middle-aged donors without and those with one or more cardiovascular and/or metabolic disease.

## DISCUSSION

In the present study, we showed that *in vitro* 53BP1 foci and TAF, but not micronuclei, are significantly positively associated with the chronological age of the donor that the fibroblasts were derived from. No association of 53BP1 foci and TAF was found within the middle-aged group dependent on the membership of a long-lived family or the presence of cardiovascular and/or metabolic disease. We found evidence for a positive association between micronuclei and 53BP1 foci.

Our results on chronological age and 53BP1 foci and TAF are in line with our previous studies that showed *in vitro* responses of fibroblasts dependent on the life histories of the donors [22,23]. Our results regarding 53BP1 foci and chronological age are in line with other studies that have observed a positive association of DNA damage foci (containing e.g. DDR mediator protein 53BP1 or phosphorylated H2AX) with chronological age in cells derived from mice [24] and in several animal tissues [3,6,25]. Unrepaired TAF also accumulate with aging in primates [4,7]. In humans, DNA damage foci are more prevalent in lymphocytes as well as primary fibroblasts of older donors compared to young [9,10]. We solidify these observations within a large group of donors and extent the available knowledge to extreme ages. Notable, the numbers of foci were higher in our study compared to the study by Waldera-Lupa et al. Since the number of foci increase towards replicative senescence [26] the higher number of foci is likely due to the higher passage number of our fibroblasts, although all strains were in early IIa stage [27]. The positive association between DNA damage foci with donor age in our study supports the role of DNA damage in human aging. Fibroblasts from donors with Werner syndrome exhibit more DNA foci compared to normal donors as well [9]. On the other hand, the donors aged 90 years have apparently reached this age despite the number of DNA damage foci, indicating that the higher numbers of DNA damage foci are a consequence of high age.

In contrast to the positive association of 53BP1 foci and TAF with chronological age, no association was found with biological donor age. This might be explained by the fact that many of the cardiovascular and/or metabolic diseases manifest around middle age, so only had little time to exert any ‘imprint’, while the differences in chronological age were much higher. Furthermore, in this relatively healthy group of middle-aged donors, the prevalence of cardiovascular and/or metabolic disease was low. Therefore, lack in contrast of the studied sample cannot be excluded. In a recent study higher numbers of micronuclei within lymphocytes of 52 patients with metabolic syndrome has been found compared to normal controls [28].

Although an inverse association of micronuclei *in vitro* has been found with maximum lifespan of different mammal species [8], we did not find a positive association between micronuclei and chronological age of the donor. In contrast, other studies have found such an association in human peripheral blood lymphocytes (age range of studies: 0-85 years) [29-32] or buccal cells (<40 compared to ≥40 years) [12]. Several explanations can be suggested: firstly, in dermal fibroblasts the baseline frequency of micronuclei is not dependent on age. Alternatively, during the cell expansion before starting the experiments, micronuclei positive fibroblasts could have been discarded or degraded their micronuclei [33]. Micronuclei can also disappear in daughter cells by being incorporated into the main nuclei [34].

We did not observe a substantial influence of rotenone on 53BP1 foci or TAF, which could be explained by its mostly aneugenic action [35,36]. As expected, stressing the fibroblasts with rotenone did results in higher numbers of micronuclei, but this higher prevalence of micronuclei in fibroblasts after rotenone incubation was also not associated with chronological donor age, similarly with previous results of human lymphocytes treated with irradiation [37,38] or mitomycin-C [39].

The strength of the study is the number of donors of a wide chronological age range. We have used several DNA damage markers in non-stressed and stressed conditions and all experiments were conducted under highly standardized conditions. One limitation of this study is the use of rotenone as stressor; other stressors could yield different results. The acquisition of biopsies and subsequent culturing of fibroblasts was done in a highly standardized manner, minimizing variation due to differences in handling. To further minimize potential random batch effects we additionally adjusted for this in our statistical analyses.

In conclusion, we showed a significant association between the prevalence of *in vitro* 53BP1 foci and TAF positive fibroblasts and the chronological age of the donor, emphasizing the link between DNA damage and human aging. However the fact that 53BP1 foci and TAF were not associated with biological age, and that micronuclei were not linked to chronological age in the present study indicates the need of further study into the influence of genome stability on *in vivo* aging. Specifically, the results of this study within primary human cells should be expanded to human aged tissues.

## METHODS

### Study design

In the prospective population-based Leiden 85-plus Study all inhabitants of Leiden (The Netherlands) aged 85 years were invited to participate [13]. A biobank of fibroblasts from skin biopsies of 68 of the surviving 90 year old donors was established from December 2003 to May 2004, as well as fibroblasts from skin biopsies of 27 young donors (18-25 years) from August to November 2006 [14]. In the Leiden Longevity Study (LLS) factors contributing to familial longevity are studied [15]. Middle-aged offspring of nonagenarian siblings were included in the study, with their age and environmentally matched partners as controls. The offspring of these families were shown to have lower mortality rates and better clinical characteristics such as fewer cases of hypertension and diabetes mellitus [16] compared to their partners. From 150 offspring-partners pairs skin biopsies were obtained and a biobank with their fibroblasts was established from November 2006 to May 2008. Both studies were approved by the Medical Ethical Committee of the Leiden University Medical Center and written informed consent was obtained from all donors.

### Characteristics of the donors

For each donor demographic characteristics were available, information on medical history was obtained from the participants' treating physicians and data on smoking habits was obtained through questionnaires. Cardiovascular and/or metabolic diseases were defined as disease history of myocardial infarction, cerebrovascular accident, hypertension or diabetes mellitus.

### Culture conditions and experimental set-up

Skin biopsies were taken from the sun-unexposed upper inner arm and fibroblast strains were cultured under predefined, highly standardized conditions as published earlier [14]. Ten strains of young donors and ten strains of old donors were randomly selected out of the Leiden 85-plus Study. Forty strains of middle-aged offspring from long-lived families and 40 strains of their partners were randomly chosen (LLS). The methods of culture conditions and experimental set-up have been described previously [17]. In short, fibroblast strains were thawed at day 0 (fibroblasts from Leiden 85-plus Study at passage 11, fibroblasts from LLS at passage 7) and subsequently passaged 3 more times over 17 days to multiply the number of fibroblasts. At day 18 the fibroblasts were seeded at 4400 cells per chamber in Permanox slides, in batches of eight strains per condition. To stress the fibroblast strains the medium (Dulbecco's Modified Eagle Medium:F-12 (1:1) medium supplemented with 10% fetal calf serum (batch no. 40G4932F), 1 mM MEM sodium pyruvate, 10 mM HEPES, 2 mM glutamax I, antibiotics (100 U/mL penicillin, 100 μg/mL streptomycin, and 0.25–2.5 μg/mL amphotericin B, all obtained from Gibco, Breda, The Netherlands) was supplemented for 72 hours with 0.6 μM rotenone (Sigma, St Louis, MO). Each experiment was performed in duplicate. At day 21 fibroblasts were fixed with 4% paraformaldehyde in ice-cold PBS for 20 minutes and washed 3 times with ice-cold PBS. The samples were subsequently stored at 2-8°C before further analysis.

### Detection of DNA damage markers: 53BP1 foci, TAF and micronuclei

Fibroblasts were permeabilized with 0.2% Triton X-100 (Sigma, St Louis, MO, USA) in PBS (PBST-0.2%) during a 20 minute incubation. Fibroblasts were further washed for 5 minutes with PBS 3 times and then covered for 1 hour with blocking buffer (4% BSA in PBST-0.1%). Next, fibroblasts were incubated with primary Rabbit anti 53BP1 antibodies (Novus Biologicals LLC, Littleton, CO, USA) diluted to a 1:1000 concentration in blocking buffer for 2 hours in humidified chambers at room temperature. Fibroblasts were washed 3 times for 15 minutes with PBST-0.1% before incubation for 1 hour with Alexa 488-conjugated Goat anti-rabbit antibodies (Invitrogen, Breda, The Netherlands) diluted to a 1:1000 concentration in blocking buffer. After 3 times 15 minute washing steps with PBST-0.1%, the secondary antibodies were cross-linked to the sample with 20 minute incubation with 4% paraformaldehyde in PBS. Fibroblasts were washed 3 times for 5 minutes with PBS and then dehydrated by covering them with increasingly higher concentrations of ethanol (70%, 80% and 95%) at a 3 minute duration per step and then air-dried. Nuclear DNA was denatured in hybridization buffer containing 0.5 μg /ml (C3TA2)3-Cy3-labeled Peptide Nucleic Acid (PNA) telomeric probe (Panagene Inc, Daejeon, South Korea) at 85° C for 5 minutes. Afterwards fibroblasts were further incubated overnight in the same buffer at room temperature and in the dark. On the following day samples were washed twice with 70 % formamide/0.67 x SSC (0.3 M NaCl, 30 mM Na_3_citrate x 2H_2_O, pH=7), followed by a 10 minute wash with 2 x SSC and a 10 minute wash with PBS. The fibroblasts were then incubated for 1 hour with Donkey anti-goat alexa-488 antibodies (Invitrogen, Breda, the Netherlands) diluted to a 1:1000 concentration in blocking buffer. The samples were washed 3 times for 15 minutes with PBST-0.1, rinsed with distilled water. The samples were then mounted with DAPI containing Prolong Gold antifade mounting medium (Invitrogen, Breda, the Netherlands).

Photographs of the samples were taken with a Leica DM5500 B microscope (Leica Microsystems, Rijswijk, the Netherlands). 53BP1 foci and micronuclei per nucleus were counted manually and automatically, which yielded consistent results at low counts, but measurements diverged at higher counts. Overall, manual and automatic counts were significantly correlated, with coefficients >0.5, thus manual counts of 53BP1 foci and micronuclei were chosen for the subsequent analysis.

Per individual donor on average 114 nuclei (rotenone-stressed state) and 124 nuclei (non-stressed state) were scored for 53BP1 foci and micronuclei. Clearly identifiable glaring dots inside the nucleus were manually counted as 53BP1 foci. Micronuclei were scored in nuclei whose surrounding area was entirely visible. We excluded from the count all nuclei lying at the edge of the image, eliminating the risk of underestimating micronuclei. Micronuclei, morphologically identical but smaller than the main nucleus, were scored according to the following characteristics as described previously [18]: 1) the diameter of micronuclei should be 1/16^th^ to 1/3^rd^ of the mean diameter of the main nucleus; 2) micronuclei should not be linked or connected to the main nucleus; 3) micronuclei may touch but not overlap the main nucleus and the micronuclear boundary should be distinguishable from the nuclear boundary. Micronuclei usually have the same staining intensity as the main nuclei, but occasionally staining may be more intense. For telomere-associated foci 100 randomly selected nuclei per donor were automatically scored for the number of 53BP1 foci together with the number of visible telomeres by using the program Stacks [19-21] which enabled to exclude the background from analysis. All counts were performed in blind with respect to donor age and offspring or partner origin.

### Statistics

All analyses were performed using IBM SPSS Statistics 20 software package. As most nuclei had few foci and micronuclei, the data distribution was skewed, thus foci and micronuclei counts are presented as the percentage of nuclei with ≥1 and ≥2 53BP1 foci/TAF per nucleus, and percentage of cells with ≥1 and ≥2 micronuclei per cell. Analogous to an earlier study [9] we also computed the percentage of nuclei with ≥3 53BP1 foci/TAF per nucleus and the percentage of cells with ≥3 micronuclei per cell, however these were very low hindering further comparison. We thus used 2 thresholds in this study: the minimum threshold of ≥1 and a threshold of ≥2. Analyses of Table 2 were performed by using linear mixed models, taking the repeated measurements of duplicate experiments into account with the covariance structure compound symmetry. Adjustments were made for random batch effects and for gender. For the supplementary analysis on whole-genome 53BP1 foci and telomere associated foci dependent on long-lived family member status a second model was used that additionally adjusted for chronological age. In the supplementary analyses on whole-genome 53BP1 foci and telomere associated foci dependent on the presence of cardiovascular or metabolic diseases, this second model consisted of additional adjustments for chronological age and long-lived family member status. A chi-square for linearity (linear by linear test) was performed in the association between micronuclei counts and 53BP1 foci. Absolute micronuclei counts (of both duplicate experiments) were linked to the presence of 53BP1 foci within the same cell. Due to the low number of high micronuclei counts, all micronuclei counts equal to or higher than 3 were combined in one category.

## SUPPLEMENTARY TABLES


