# Supplementary material for: DNA damage markers in dermal fibroblasts in vitro reflect chronological donor age
Source: Aging (Albany NY). 2016 Jan 30;8(1):147–55. doi: 10.18632/aging.100890 (PMC4761719; doi:10.18632/aging.100890)
Supplement: Supplementary file 1 [file aging-08-147-s001.pdf]

SUPPLEMENTARY TABLES

Supplementary Table 1. 53BP1 foci, TAF and micronuclei dependent on membership of a long-lived family.

	Familial longevity		
	Offspring		
	N=40	N=40	P-value
**Non-stressed state**			
% nuclei with ≥1 53BP1 foci/nucleus	47.3 (2.90)	48.1 (2.90)	0.730
% nuclei with ≥2 53BP1 foci/nucleus	13.9 (1.83)	14.7 (1.83)	0.565
% nuclei with ≥1 TAF/nucleus	23.0 (1.62)	26.3 (1.62)	0.070
% nuclei with ≥2 TAF/nucleus	5.18 (1.12)	5.43 (1.13)	0.862
% cells with ≥1 micronuclei/cell	1.05 (0.17)	0.92 (0.14)	0.606
% cells with ≥2 micronuclei/cell	0.09 (0.04)	0.10 (0.04)	0.897
**Rotenone-stressed state**			
% nuclei with ≥1 53BP1 foci/nucleus	47.8 (3.04)	49.4 (3.05)	0.487
% nuclei with ≥2 53BP1 foci/nucleus	19.3 (2.67)	20.5 (2.68)	0.543
% nuclei with ≥1 TAF/nucleus	21.4 (1.49)	21.9 (1.51)	0.748
% nuclei with ≥2 TAF/nucleus	3.97 (0.54)	4.76 (0.55)	0.232
% cells with ≥1 micronuclei/cell	12.1 (0.61)	10.9 (0.63)	0.198
% cells with ≥2 micronuclei/cell	2.06 (0.18)	1.81 (0.19)	0.343
**Δ stressed and non-stressed state**			
% nuclei with ≥1 53BP1 foci/nucleus	0.24 (2.96)	1.27 (2.97)	0.586
% nuclei with ≥2 53BP1 foci/nucleus	5.44 (1.94)	5.96 (1.95)	0.737
% nuclei with ≥1 TAF/nucleus	-1.50 (1.15)	-4.01 (1.18)	0.088
% nuclei with ≥2 TAF/nucleus	-1.09 (0.94)	-0.62 (0.97)	0.728
% cells with ≥1 micronuclei/cell	11.0 (0.63)	10.1 (0.66)	0.295
% cells with ≥2 micronuclei/cell	1.96 (0.18)	1.71 (0.19)	0.332

The adjusted estimated means within offspring and partners are given (linear mixed model, adjustment for gender, age, batch and repeated measurements). SE: standard error, Δ: difference.

Supplementary Table 2. 53BP1 foci, TAF and micronuclei dependent on presence of cardiovascular and/or metabolic diseases.

	Cardiovascular and/or metabolic diseases			
	Absent N=52	Present N=21	P for trend[a]	P for trend[b]
**Non-stressed state**				
% nuclei with ≥1 53BP1 foci/nucleus	47.0 (2.60)	46.5 (3.11)	0.847	0.487
% nuclei with ≥2 53BP1 foci/nucleus	13.8 (1.38)	13.1 (1.69)	0.621	0.493
% nuclei with ≥1 TAF/nucleus	24.6 (1.48)	23.3 (2.07)	0.589	0.216
% nuclei with ≥2 TAF/nucleus	4.80 (1.00)	6.06 (1.46)	0.453	0.337
% cells with ≥1 micronuclei/cell	1.07 (0.16)	0.95 (0.24)	0.689	0.562
% cells with ≥2 micronuclei/cell	0.11 (0.03)	0.08 (0.05)	0.632	0.764
**Rotenone-stressed state**				
% nuclei with ≥1 53BP1 foci/nucleus	47.1 (2.78)	48.2 (3.40)	0.729	0.775
% nuclei with ≥2 53BP1 foci/nucleus	18.7 (2.35)	19.6 (2.87)	0.726	0.849
% nuclei with ≥1 TAF/nucleus	21.7 (1.52)	20.2 (1.89)	0.399	0.174
% nuclei with ≥2 TAF/nucleus	4.48 (0.53)	3.49 (0.71)	0.193	0.077
% cells with ≥1 micronuclei/cell	11.6 (0.58)	11.6 (0.86)	0.977	0.358
% cells with ≥2 micronuclei/cell	1.97 (0.17)	1.91 (0.25)	0.857	0.992
**Δ stressed and non-stressed state**				
% nuclei with ≥1 53BP1 foci/nucleus	-0.01 (2.86)	1.49 (3.25)	0.537	0.675
% nuclei with ≥2 53BP1 foci/nucleus	5.00 (1.88)	6.55 (2.28)	0.439	0.805
% nuclei with ≥1 TAF/nucleus	-2.37 (1.09)	-3.08 (1.55)	0.690	0.851
% nuclei with ≥2 TAF/nucleus	-0.22 (0.88)	-2.45 (1.31)	0.161	0.067
% cells with ≥1 micronuclei/cell	10.6 (0.60)	10.6 (0.90)	0.961	0.339
% cells with ≥2 micronuclei/cell	1.85 (0.17)	1.83 (0.25)	0.940	0.864

Only donors from the LLS were included in this analysis. The adjusted estimated means within both groups (disease absent or present) are given. Linear mixed model: model 1 ([a]) adjustment for gender, batch and repeated measurements. Model 2 ([b]): as model 1 plus adjustment for age and long-lived family member status. SE: standard error, Δ: difference.
